# Involvement of PPARs in Cell Proliferation and Apoptosis in Human Colon Cancer Specimens and in Normal and Cancer Cell Lines

**DOI:** 10.1155/2007/93416

**Published:** 2007-03-13

**Authors:** G. Martinasso, M. Oraldi, A. Trombetta, M. Maggiora, O. Bertetto, R. A. Canuto, G. Muzio

**Affiliations:** Department of Experimental Medicine and Oncology, University of Turin, Corso Raffaello 30, 10125 Turin, Italy

## Abstract

PPAR involvement in cell growth was investigated “in vivo” and “in vitro” and was correlated with cell proliferation and apoptotic death. “In vivo” PPAR*γ* and *α* were evaluated in colon cancer specimens and adjacent nonneoplastic colonic mucosa. PPAR*γ* increased in most cancer specimens versus mucosa, with a decrease in c-Myc and in PCNA proteins, suggesting that colon cancer growth is due to increased cell survival rather than increased proliferation. The prevalence of survival over proliferation was confirmed by Bcl-2 or Bcl-X_*L*_ increase in cancer versus mucosa, and by decreased PPAR*α*. “In vitro” PPAR*γ* and PPAR*α* were evaluated in human tumor and normal cell lines, treated with natural or synthetic ligands. PPAR*γ* was involved in inhibiting cell proliferation with a decrease in c-Myc protein, whereas PPAR*α* was involved in inducing apoptosis with modulation of Bcl-2 and Bad proteins. This involvement was confirmed using specific antagonists of two PPARs. Moreover, the results obtained on treating cell lines with PPAR ligands confirm observations in colon cancer: there is an inverse correlation between PPAR*α* and Bcl-2 and between PPAR*γ* and c-Myc.

## 1. INTRODUCTION

Nuclear receptors are attractive targets for novel therapeutic approaches to cancer. Treating malignant tumors by inducing cell differentiation is an attractive concept, but the clinical development of differentiation-inducing agents to treat malignant tumors, especially for solid tumors, has been limited to date [1].

Among the large group of nuclear receptors, a special place is occupied by peroxisome proliferator-activated receptors (PPARs), which are involved in controlling metabolism, growth, and immune and inflammatory responses [2]. In addition to these metabolic and anti-inflammatory properties, PPARs have been indicated as both tumor suppressors and tumor promoters [3, 4]. Upon activation by specific agonists, these receptors form dimers with RXR receptors and translocate to the nucleus, where they act as agonist-dependent transcription factors and regulate gene expression by binding to specific promoter regions of target genes [5, 6].

PPAR*γ* is expressed in many cancers, including colon, breast, and prostate, and specific PPAR*γ* agonists, such as thiazolidinediones, are generally considered antiproliferative in these settings [7]. However, although PPAR*γ* acts as a tumor suppressor in colon cancer, colon tumors with mutations in the APC gene appear to be exceptions, since thiazolidinediones promote growth in these tumors [8, 9].

Less is known about the role of PPAR*α* in human tumors. Generally, activation of this PPAR by exogenous agonists causes inhibition of tumor cell growth [10–12]. Among specific agonists of PPAR*α*, fibrates, a widely used class of hypolipidemic drugs, act by modulating transcription of genes encoding proteins that control lipid transport and metabolism, and also exert pleiotropic anti-inflammatory effects by downregulating the expression of genes encoding inflammatory cytokines and acute-phase response proteins [13]. These combined actions translate into clinical benefits, as demonstrated by reduced cardiovascular morbidity and mortality in primary and secondary intervention trials [14].

The physiological and pharmacological roles of PPAR*β* (also referred to as PPAR*δ*) are just beginning to emerge. It has recently become clear that PPAR*β* has a function in epithelial tissues, but inconsistent reports leave the situation controversial. There is strong evidence that activation of PPAR*β* by ligands such as di(2-ethylhexyl) phthalate can induce terminal differentiation of keratinocytes, with concomitant inhibition of cell proliferation [15, 16]. However, the role of PPAR*β* in keratinocyte-specific apoptosis is less clear, and its role in colonic epithelium is also unclear due to conflicting evidence suggesting that its ligand activation can both potentiate and attenuate intestinal cancer [16].

PPAR isoforms are receptors for several ligands, some of which are natural, such as PUFAs and their derivatives, while others are synthetic, such as the above-mentioned fibrates and phthalates. The ligands have different affinities for the receptors. All three isoforms can be activated by *n*-3 and *n*-6 PUFAs, although the affinity of PUFAs for the receptors varies, which suggests a role for site-specific availability and metabolism of particular fatty acids and differences in their affinities for specific PPAR subtypes [17].

Our interest is to demonstrate the different involvements of PPARs in cell growth, with particular attention to the carcinogenic process, and to correlate them to cell proliferation and death by apoptosis. Investigations have been carried out both “in vivo” and “in vitro.” “In vivo” PPAR protein content was evaluated in several colon cancer specimens from patients undergoing surgery to remove tumors. “In vitro” PPAR protein content was evaluated in the human lung-tumor A549 cell line and in the normal human proliferating NCTC 2544 cell line. These two cell lines were chosen because the first does not express PPAR*α*, whereas the second does not express PPAR*γ*. They thus appeared to be a good choice to show the importance of PPAR*γ* and PPAR*α* in cell proliferation and apoptosis. To induce PPARs, docosahexaenoic acid (DHA) was chosen as ligand for PPAR*γ* and clofibrate for PPAR*α*.

## 2. MATERIALS AND METHODS

### 2.1. Colon tissue samples

Specimens of colon cancer and adjacent nonneoplastic colonic mucosa were obtained from surgically excised material of 24 patients at the Oncological Center, Molinette Hospital, Turin, Italy. Adjacent connective tissue was in all cases stripped to eliminate fat contamination, and colonic cancer specimens were without nonneoplastic colon mucosa; specimens were immediately frozen and stored at −80°C.

### 2.2. Culture conditions

A549 human lung adenocarcinoma cells (ATCC, USA) were seeded (25 000 cells/cm^2^) and maintained for 24 hours in Ham's F-12K medium supplemented with 2 mM glutamine, 1% (v/v) antibiotic/antimycotic solution (medium A), and 10% (v/v) fetal bovine serum (FBS). Human keratinocyte NCTC 2544 cells (kind gift from Dr.Bassi, University of Genoa, Italy) were seeded (20 000 cells/cm^2^) in DMEM (low glucose medium) plus 2 mM glutamine, 1% (v/v) antibiotic/antimycotic solution, 1% nonessential amino acids, and 10% (v/v) fetal bovine serum. All cells were maintained at 37°C in a humidified atmosphere of 5% CO_2_ in air.

### 2.3. Treatment of A549 cells with docosahexaenoic acid

A stock solution of free fatty acid (100 mM in FBS) was prepared and stored at −20°C until use. Fatty acid was purchased from Sigma Chemical Co. (St. Louis, Mo).

Twenty four hours after cell seeding (time 0), culture medium was removed and replaced by medium B (medium A plus 0.4% serum bovine albumin (fatty-acid free), 1% ITS (insulin, transferrin, sodium selenite), 1% vitamin solution) supplemented or not with DHA. Stock solution of DHA was diluted to the final concentrations, reported in the figures, directly in medium B, used to replace medium A. FBS was added to control cells.

Forty eight hours after DHA addition, treated and control cells were trypsinized after collecting culture media, harvested and centrifuged at 600 g for 10 minutes to carry out the assays listed below.

### 2.4. Treatment of A549 cells with docosahexaenoic acid and PPAR*γ* antagonist

Twenty four hours after cell seeding (time 0), culture medium was removed and replaced by medium B supplemented with 50 *μ*M DHA and 10 *μ*M GW9662. The cells were processed as explained above.

### 2.5. Treatment of NCTC 2544 with clofibrate

Twenty four hours after cell seeding, clofibrate dissolved in DMSO (maximum final concentration 0.05%) was added to cells at the concentrations reported in the figures. Solvent alone was added to control cells. At 24- or 48-hour treatment, the cells were trypsinized after collecting culture media, and centrifuged at 600 g for 10 minutes. Cells and culture media were used for the assays listed below.

### 2.6. Treatment of NCTC 2544 with clofibrate and PPAR*α* antagonist

Twenty four hours after cell seeding, 250 *μ*M clofibrate and 5 *μ*M MK886 were added to cells. The cells were processed as explained above.

### 2.7. Cell proliferation

Cell proliferation was determined as by Martinasso et al. [15].

### 2.8. Apoptosis

Apoptosis was determined by evaluating the presence of a sub-G0/G1 peak in flow cytometric analysis as by Martinasso et al. [15], and by TUNEL staining. For flow cytometric analysis, briefly, cells were washed in PBS, fixed in ice-cold 70% ethanol for at least 30 minutes, incubated at room temperature in PBS containing DNase-free RNase (type II-A) and propidium iodide, respectively, at final concentrations of 0.4 and 0.18 mg/mL, and then analyzed with a FACScan flow cytometer (Becton & Dickinson, Mountain View, Calif, USA) equipped with a 488 nm argon laser and 2 filters, transmitting at 585 nm (FL2) and above 620 nm (FL3), respectively. Data were recorded on a Hewlett Packard computer (HP 9000, model 300), using CellFit software (Becton & Dickinson).

TUNEL staining was performed using the DeadEnd Colorimetric TUNEL System (Promega, Madison, Wis, USA) following the manufacturer's instructions.

### 2.9. Western blot analysis

For each specimen, about 50 mg of tissue were washed twice in cold PBS and then homogenized by sonication in 150 *μ*L of lysis buffer (Totex buffer (pH 7.9) containing 20 mM Hepes, 350 mM NaCl, 20% glycerol, 1% NP-40 substitute, 1 mM MgCl_2_, 0.5 mM EDTA, 0.1 mM EGTA, 1 mM Na-orthovanadate, 1 mM phenyl methyl sulfonyl fluoride, 15 *μ*g/mL leupeptin). The homogenates were kept on ice for 1 hour and then centrifuged in a microfuge at 13 500 rpm, for 25 minutes. Collected cells were washed twice in cold PBS, suspended to 50% (w/v) in the same lysis buffer, incubated in ice for 30 minutes, and sonicated. The homogenates were centrifuged in a microfuge at 12 000 rpm, for 10 minutes.

The supernatants containing the extracted proteins were then collected and used for Western blot analysis.

After SDS-polyacrylamide gel electrophoresis, the proteins were electrotransferred to a PVDF membrane, which was then blocked overnight with TBS containing 10% nonfat dried milk. The membranes were then rinsed and incubated with polyclonal anti-PPAR*γ*, anti-PPAR*α*, anti-PPAR*β*, anti-PCNA (proliferating cell nuclear antigen), anti-Bcl-2, or anti-Bcl-X_*L*_ antibodies, with monoclonal anti-Bad, anti-c-Myc (all from Santa Cruz Biotechnology, Germany), or anti-*β*-actin antibodies (from Sigma Chemical Co., St. Louis, Mo, USA). Protein bands were visualized using a chemiluminescent detection system (Immun-Star HRP, Bio-Rad, Calif, USA).

### 2.10. Protein determination

Protein content was determined with the Protein Assay Kit 2 (Bio-Rad).

### 2.11. Statistical analysis

All data are expressed as means ± SD. The significance of differences between group means was assessed by variance analysis, followed by the Newman-Keuls test or by the student *t* test.

## 3. RESULTS

### 3.1. PPAR*γ* and PPAR*α* protein content in colon cancers

**Figure 1:**
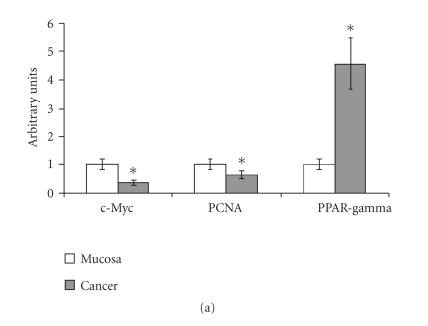

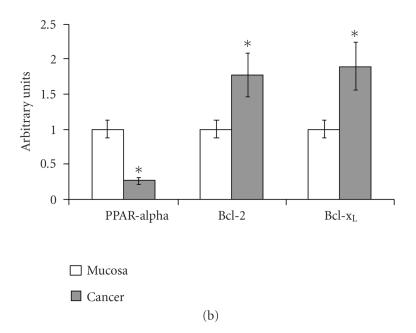
PPAR*γ*, c-Myc, PCNA, PPAR*α*, Bcl-2, and Bcl-X_*L*_ protein contents in colon cancer specimens.Values are means ± SD of 19 patients for PPAR*γ*, 20 patients for PCNA and c-Myc (a), 21 patients for PPAR*α*, 13 patients for Bcl-2, and 18 patients for Bcl-X_*L*_ (b). The densitometry value of each protein for each patient was normalized to the corresponding *β*-actin value and was expressed as related to that of the corresponding mucosa specimen, set arbitrarily to 1. ∗ *t* test, colon cancer versus nonneoplastic mucosa (*P* < .05).

“In vivo” PPAR*γ* and PPAR*α* proteins content was evaluated in cancer specimens from patients undergoing surgery to remove colon tumors. Samples of adjacent nonneoplastic colonic mucosa from the same patients were also collected for comparison. In 19 of 24 patients, PPAR*γ* protein content was higher in cancer specimens than in mucosa specimens, showing that PPAR*γ* was generally increased in cancer versus mucosa (Figure 1(a)). Protein contents in each mucosa specimen were set arbitrarily to 1, and values relating to tumor specimens referred to the corresponding mucosa specimen.

The PPAR*γ* increase was coupled with a decrease in c-Myc and in PCNA (Figure 1(a)), suggesting that colon cancer growth is due to increased survival rather than due to increased cell proliferation. In 20 of 24 patients, the protein content of c-Myc and of PCNA in cancer specimens was above that in mucosa specimens. The prevalence of cell survival over cell proliferation was confirmed by the increase of Bcl-2 or Bcl-X_*L*_, both antiapoptotic proteins, in cancer versus mucosa specimens, and by the decrease of PPAR*α* (Figure 1(b)). PPAR*α* was decreased in 21 patients, Bcl-2 was increased in 13 patients, and Bcl-X_*L*_ was increased in 18 patients of 24 patients. Only in 2 patients were both Bcl-2 and Bcl-X_*L*_ decreased. In all patients, an inverse correlation between PPAR*α* and antiapoptotic proteins was observed. No univocal changes were observed for PPAR*β* (data not reported).

### 3.2. Apoptosis in colon cancers

**Figure 2:**
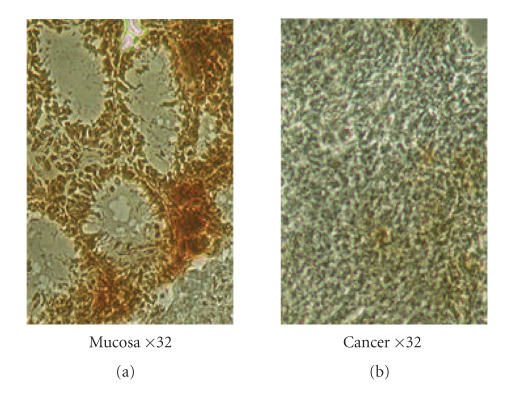
Apoptosis evaluated in mucosa and colon cancer by TUNEL staining.

Apoptosis was evaluated by TUNEL staining, as shown in Figure 2. In nonneoplastic colonic mucosa, a large number of TUNEL-positive nuclei were evident, whereas in cancer specimens the number of TUNEL-positive nuclei was lower than in corresponding mucosa.

### 3.3. PPAR protein contents in human cell lines

“In vitro” PPAR*γ*, *β* and *α* protein contents were evaluated in tumor (lung adenocarcinoma A549) and normal (keratinocytes NCTC 2544) human cell lines, treated with natural or synthetic ligands (DHA or clofibrate). DHA was used as ligand of PPAR*γ*, whereas clofibrate was used as specific ligand of PPAR*α*.

**Figure 3:**
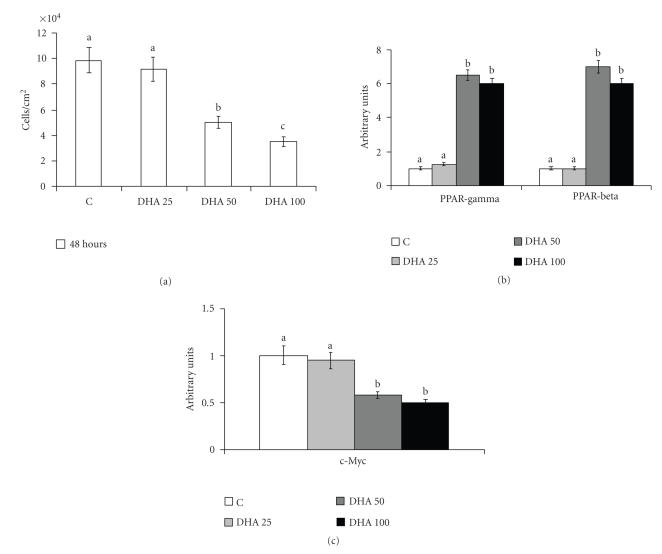
Cell growth, and PPAR*γ*, PPAR*β*, and c-Myc protein contents, in human lung tumor A549 cells treated with DHA. (a) Cell growth: 24 hours after seeding, cells were treated with 50 *μ*M of DHA for a further 48 hours. Control cells were treated with vehicle alone. The number of cells/cm^2^ counted in the monolayer is represented as mean ± SD of 3 experiments. (b) Protein content of PPAR*γ*, PPAR*β*, and (c) c-Myc: protein content was determined by Western blot analysis. The densitometry value given for each protein referred to the corresponding *β*-actin value and was expressed by arbitrarily normalizing the control value to 1. Means with different letters are significantly different from one another (*P* < .05) as determined by analysis of variance followed by post hoc Newman-Keuls analysis.

Figure 3 shows the increase of protein content of PPAR*γ* inversely correlated with the inhibition of cell proliferation, as evidenced by the decrease of cell numbers and of c-Myc in cells treated with PUFA at different concentrations for 48 hours in comparison with control cells. The protein content of PPAR*γ* and c-Myc in treated cells referred to content of control cells, set arbitrarily to 1. No cells in apoptosis or in necrosis were found after treating A549 cells with this ligand (data not shown). With regard to PPAR*β* and *α* protein contents, in A549 cells only PPAR*β* was increased, as it is also shown in Figure 3(b), since PPAR*α* was not expressed (data not shown).

PPAR*γ* was also found to be involved in the signal transduction pathway inhibiting cell proliferation, for example in HepG2 treated with CLA and in A549 treated with arachidonic acid [18, 19].

**Figure 4:**
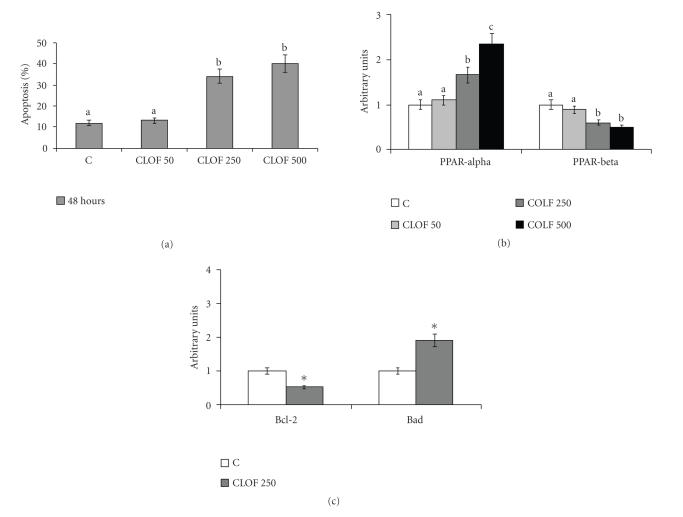
Apoptosis, and PPAR*α*, PPAR*β*, Bcl-2, and Bad protein contents, in human keratinocytes NCTC 2544 treated with clofibrate. (a) Apoptosis: 24 hours after seeding, cells were treated with 250 *μ*M clofibrate (CLOF) for a further 48 hours. Control cells were treated with vehicle alone. Apoptosis was determined by flow cytometric analysis. The percentages of apoptotic cells are presented as means ± SD of 3 experiments. (b) and (c) Protein content of PPAR*α*, PPAR*β*, Bcl-2, and Bad: protein content was determined by Western blot analysis. The densitometry value given for each protein and for each patient referred to the corresponding *β*-actin value and was expressed by arbitrarily normalizing the control value to 1. Means with different letters are significantly different from one another (*P* < .05) as determined by analysis of variance followed by post hoc Newman-Keuls analysis. ∗ *t* test, treated cells versus untreated cells (*P* < .05).

PPAR*α* has been reported to be involved in the signal transduction pathway inducing apoptosis [18, 20]. As Figure 4 shows, PPAR*α* protein content (Figure 4(b)) was increased in NCTC 2544 cells treated with clofibrate, at different concentrations for 48 hours, in comparison with control cells; apoptosis increased in parallel (Figure 4(a)). PPAR*γ* and *β* were also evaluated: the former was not expressed, whereas the latter (Figure 4(b)) was decreased by treatment with clofibrate.

**Figure 5:**
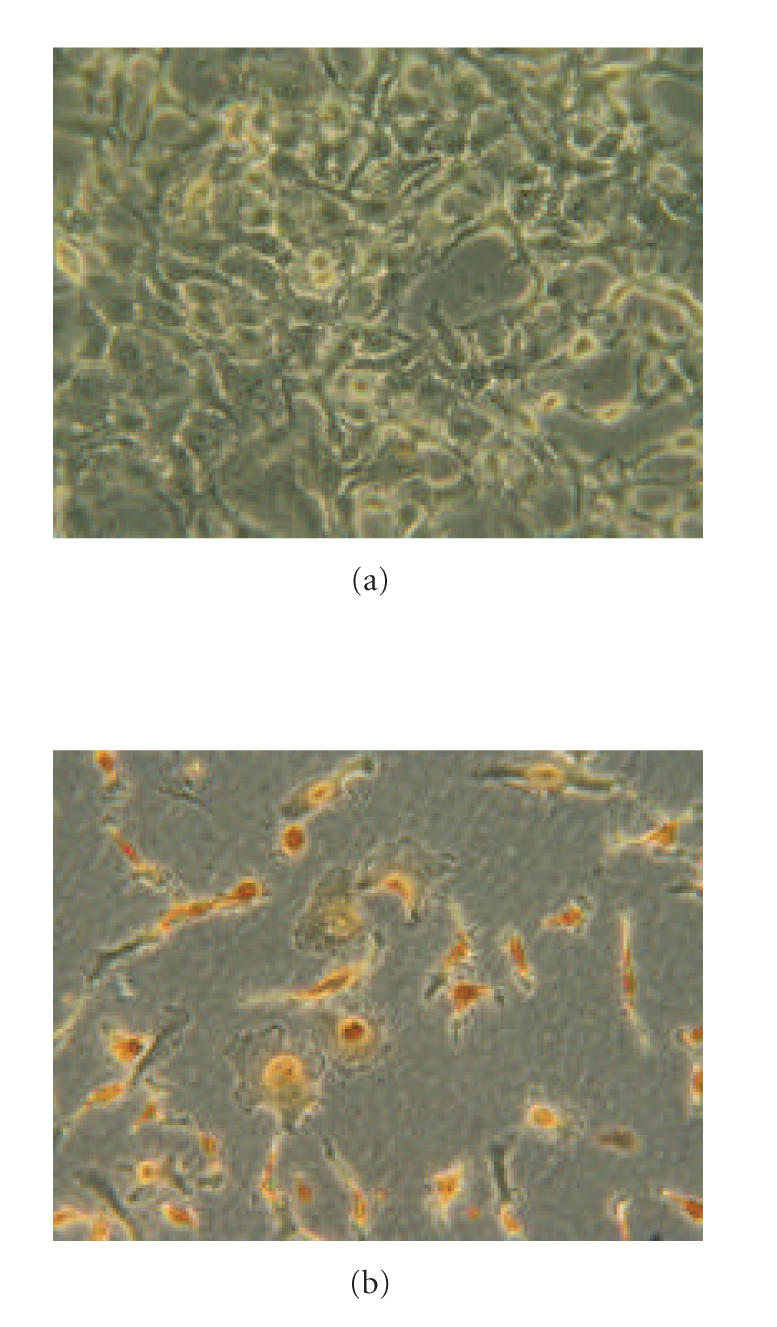
Apoptosis in human keratinocytes NCTC 2544 treated with clofibrate. Twenty four hours after seeding, cells were treated with 250 *μ*M clofibrate for a further 48 hrs. Control cells were treated with vehicle alone. Apoptosis was determined by TUNEL staining. Panel A, control cells; Panel B, cells treated with clofibrate.

Apoptosis was evaluated by flow cytofluorimetric analysis, and TUNEL staining was also carried out to confirm the presence of cells in apoptosis. Figure 5 shows that NCTC 2544 cells treated with 250 *μ*M clofibrate were positive to TUNEL staining, indicating DNA fragmentation (Figure 5(b)); untreated cells (Figure 5(a)) were all negative to TUNEL staining.

The increase in PPAR*α* observed in NCTC 2544 cells (Figure 4) was accompanied by a decrease in Bcl-2 and by an increase in Bad (Figure 4(c)). The content of the different proteins in treated cells referred to the corresponding content in control cells, set arbitrarily to 1.

The concentrations of PUFA and fibrates were shown not to be cytotoxic, by determining the activity of lactate dehydrogenases in the medium; no increase of enzyme release occurred (data not shown).

### 3.4. Effect of PPAR*γ* or PPAR*α* antagonist on cell proliferation or apoptosis

To demonstrate the involvement of PPARs in cell proliferation or apoptosis, a specific antagonist for each PPAR studied was added to cells at the same time as the PPAR ligand.

**Figure 6:**
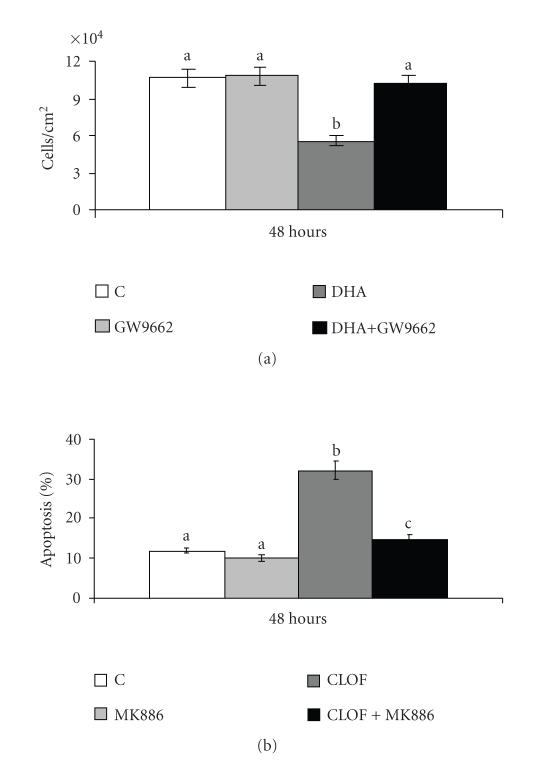
Cell growth and apoptosis, respectively, in A549 and NCTC 2544 cells after treatment with antagonist and ligand of PPARs. (a) Cell growth in A549 cells: 24 hours after seeding, cells were treated with 50 *μ*M of DHA and/or 10 *μ*M GW9662 for a further 48 hours. Control cells were treated with vehicle alone. The number of cells/cm^2^ counted in the monolayer is represented as mean ± SD of 3 experiments. (b) Apoptosis in NCTC 2544 cells: 24 hours after seeding, cells were treated with 250 *μ*M clofibrate (CLOF) and/or 5 *μ*M MK886 for a further 48 hours. Control cells were treated with vehicle alone. Apoptosis was determined by flow cytometric analysis. The percentages of apoptotic cells are presented as means ± SD of 3 experiments. Means with different letters are significantly different from one another (*P* < .05) as determined by analysis of variance followed by post hoc Newman-Keuls analysis.

Figure 6 shows that the PPAR*γ* antagonist, GW9662, completely prevented the inhibition induced by DHA on A549 cell proliferation (Figure 6(a)), and that the PPAR*α* antagonist, MK886, almost completely prevented the induction of apoptosis induced by clofibrate in NCTC 2544 cells (Figure 6(b)).

## 4. DISCUSSION

PPARs are transcription factors that may be involved in the modulation of cell proliferation and apoptosis; to strengthen this observation, their expression was evaluated “in vivo” in colon cancer and “in vitro” in human lung-tumor A549 cells and keratinocytes NCTC 2544. These cell lines were chosen, having previously been shown to lack, respectively, PPAR*α* expression and PPAR*γ* expression.

From our observations, it appears that PPAR*γ* is correlated with an inhibition of cell proliferation that is not accompanied by any increase of apoptosis: in specimens of colon cancer from patients undergoing surgery, the expression of PPAR*γ* was higher than in mucosa specimens. Together with this increase, reduced c-Myc and PCNA protein contents were observed, indicating lower cell proliferation in tumor than in mucosa specimens. PPAR*γ* is overexpressed not only in colon cancer but also in other tumor types, such as in primary human lung tumors. The increased expression of PPAR*γ* in these tumors versus the corresponding normal tissue has been demonstrated both by immunochemical staining and by Western blotting [21].

An increased PPAR*γ* expression has also been reported in lung-tumor A549 cells, when cell proliferation was reduced by treating them with the PPAR ligand, DHA. Also in this case, the increase was accompanied by a reduction of c-Myc content compared to controls. At the PUFA concentrations used, neither necrosis nor apoptosis was evidenced. In these A549 cells treated with PUFA, the increase in PPAR*γ* expression was paralleled by an increase in PPAR*β*, possibly indicating that this PPAR is also important, whereas the modification of PPAR*α* was not significant, since it failed to occur in this type of cell.

In previous work, we reported the involvement of PPAR*γ* in tumor-cell proliferation and differentiation in hepatoma and lung tumor cells [18, 19, 22–24]. Other groups have also determined this involvement: exposure of cultured human colorectal cancer cell lines to PPAR*γ* agonists inhibits growth, associated with G1 cell cycle arrest, and it increases several markers of differentiation [25, 26]. Moreover, resveratrol has also been found to inhibit cell growth of both Caco-2 and HCT-116 cells in a dose- and time-dependent manners, inducing PPAR*γ* and p38 MAPK [27]. Similarly, treatment of lung adenocarcinoma cells (A549) with troglitazone inhibits cell growth in a dose-dependent manner, due to inhibited cell proliferation [28]. The cell cycle profile reveals an arrest at G0-G1 with a concomitant downregulation of G0-G1 cyclins D and E. Similar to troglitazone, nonsteroidal anti-inflammatory drugs (NSAIDs) mediate cyclooxygenase-independent inhibition of lung cancer cell growth through PPAR*γ* activation [29].

Unlike our work, others have found that PPAR*γ* activation not only inhibits lung-cancer cell growth, increases cell differentiation, and induces cell-cycle arrest, but that it also induces apoptosis [30].

With regard to PPAR*α*, the results reported here show that changes in the expression of this transcription factor are probably involved in modulating apoptosis. Determination of PPAR*α* “in vivo” in specimens of colon cancer showed it to be decreased in comparison with mucosa specimens, alongside an increase in the antiapoptotic proteins Bcl-2 and Bcl-X_*L*_. It can be confirmed that the PPAR*α* decrease and the antiapoptotic-protein increase are involved in reducing apoptosis by determining cells in apoptosis through TUNEL staining; this showed fewer stained nuclei in cancer specimens than in the corresponding mucosa. Other authors have also reported a significant decrease in PPAR*α* expression in human tubular adenomas compared to normal human colonic epithelial cells, and this observation has raised interest in investigating PPAR*α* as a therapeutic target to prevent adenoma formation [31]. PPAR*α* and PPAR*γ* ligands have also been shown to play a potential role in suppressing both hyperlipidemia and polyp formation in Apc gene-deficient mice, an animal model for human familial adenomatous polyposis [28].

To strengthen the hypothesis of the involvement of PPAR*α* in apoptosis, tests were run on keratinocytes NCTC 2544, since they lack PPAR*γ*. The specific PPAR*α* ligand, clofibrate, induced apoptosis in these cells, the effect being accompanied by PPAR*α* induction and modulation of apoptotic proteins.

In the tumor cell line SK-HEP-1 treated with PPAR*α* ligands, such as CLA or Wy-14643, apoptosis was also induced accompanied by PPAR*α* induction, Bcl-2 decrease, and Bad increase (see [18] and other data submitted for publication). Fibrates have also been demonstrated to induce apoptosis in glioblastoma cell lines [32] and in other hepatoma cells [33].

On the contrary, other studies have shown that activation of PPAR*α* with Wy-14643 inhibited proliferation and migration of smooth muscle cells (SMCs) rather than inducing apoptosis. These results are supported by the finding that the PPAR*α* ligand clofibrate, which is used clinically to reduce serum triglyceride levels, as well as the ligand GW7647, prevented DNA synthesis by SMCs [34].

Unlike our results, others have reported that rodents develop tumors in response to exposure to a wide range of PPAR*α* ligands, suggesting that the causal events are activation of PPAR*α*, increase of cell proliferation, and inhibition of apoptosis. However, the same study found that human cells are not susceptible to these effects [35, 36].

The induction of apoptosis in NCTC 2544 cells was accompanied, alongside the increased expression of PPAR*α*, also by decreased PPAR*β* expression. These results are in agreement with the finding that PPAR*β* is involved in cell survival [37].

The involvement of PPAR*γ* especially in cell proliferation, and of PPAR*α* especially in apoptosis, was demonstrated by using specific antagonists, which had the effect, respectively, of preventing inhibition of cell proliferation by DHA, and of preventing induction of apoptosis by clofibrate.

The reason for the different effects of PPAR ligands, that is, decrease of cell proliferation in some tumor cells inducing PPAR*γ*, or induction of apoptosis in others inducing PPAR*α*, is not yet clear. Moreover, the results obtained on treating cell lines with PPAR ligands confirm observations in colon cancer: there is an inverse correlation between PPAR*α* and Bcl-2 and between PPAR*γ* and c-Myc.
